# The creation and selection of mutations resistant to a gene drive over multiple generations in the malaria mosquito

**DOI:** 10.1371/journal.pgen.1007039

**Published:** 2017-10-04

**Authors:** Andrew M. Hammond, Kyros Kyrou, Marco Bruttini, Ace North, Roberto Galizi, Xenia Karlsson, Nace Kranjc, Francesco M. Carpi, Romina D’Aurizio, Andrea Crisanti, Tony Nolan

**Affiliations:** 1 Dept. of Life Sciences, Imperial College London, London, United Kingdom; 2 Polo d’Innovazione Genomica, Genetica e Biologia, Siena, Italy; 3 Department of Zoology, University of Oxford, Oxford, England; 4 Laboratory of Integrative Systems Medicine (LISM), Institute of Informatics and Telematics (IIT), CNR, Pisa, Italy; Institute of Science and Technology Austria (IST Austria), AUSTRIA

## Abstract

Gene drives have enormous potential for the control of insect populations of medical and agricultural relevance. By preferentially biasing their own inheritance, gene drives can rapidly introduce genetic traits even if these confer a negative fitness effect on the population. We have recently developed gene drives based on CRISPR nuclease constructs that are designed to disrupt key genes essential for female fertility in the malaria mosquito. The construct copies itself and the associated genetic disruption from one homologous chromosome to another during gamete formation, a process called homing that ensures the majority of offspring inherit the drive. Such drives have the potential to cause long-lasting, sustainable population suppression, though they are also expected to impose a large selection pressure for resistance in the mosquito. One of these population suppression gene drives showed rapid invasion of a caged population over 4 generations, establishing proof of principle for this technology. In order to assess the potential for the emergence of resistance to the gene drive in this population we allowed it to run for 25 generations and monitored the frequency of the gene drive over time. Following the initial increase of the gene drive we observed a gradual decrease in its frequency that was accompanied by the spread of small, nuclease-induced mutations at the target gene that are resistant to further cleavage and restore its functionality. Such mutations showed rates of increase consistent with positive selection in the face of the gene drive. Our findings represent the first documented example of selection for resistance to a synthetic gene drive and lead to important design recommendations and considerations in order to mitigate for resistance in future gene drive applications.

## Author summary

Gene drives are selfish genetic elements that are able to bias their own inheritance among offspring. Starting from very low frequencies they can rapidly invade a population in just a few generations, even when imposing a fitness cost. Gene drives based on the precise DNA cutting enzyme CRISPR have been shown recently to be highly efficient at copying themselves from one chromosome to the other during the process of gamete formation in mosquitoes, resulting in transmission to 99% of offspring instead of the 50% expected for a single gene copy. One proposed use for CRISPR-based gene drives is in the control of mosquitoes by designing the gene drive to target mosquito genes involved in fertility, thereby reducing their overall reproductive output and leading to population suppression. Like any intervention designed to suppress a population these gene drives are expected to select for mutations in the mosquito that are resistant to the drive and restore fertility to mosquitoes. We have analyzed the origin and selection of resistant alleles in caged populations of mosquitoes initiated with a gene drive construct targeting a female fertility gene. We find the selected alleles are in-frame insertions and deletions that are resistant to cleavage and restore female fertility. Our findings allow us to improve predictions on gene drive behaviour and to make concrete recommendations on how to improve future gene drive designs by decreasing the likelihood that they generate resistance.

## Introduction

Naturally occurring gene drives—selfish genetic elements that are able to bias their own inheritance and rapidly invade a population, even starting from very low frequencies—have inspired proposals to harness their power to spread into a population of insect disease vectors traits that manipulate their biology in ways that could suppress or eliminate disease transmission [1–4]. In particular for malaria, transmitted exclusively by mosquitoes of the *Anopheles* genus, historical gains in reducing the disease burden have been largely achieved by the correct implementation of vector control measures (residual insecticides and bed nets) [5]. Though these measures have been instrumental in substantially reducing malaria transmission, they are insufficient by themselves to eradicate the disease in the near future at the current level of investment [6]. Gene drive technology could help in developing a self-sustaining, species-specific and affordable vector control measure much needed to achieve disease eradication in the future.

Gene drives based on the activity of DNA nucleases able to recognise specific target sequences were first proposed over a decade ago and have received much attention recently due to the advent of new, easily programmable nucleases such as CRISPR-Cas9 that have allowed us and others to build functioning gene drives that show rates of inheritance from a heterozygous parent close to 100%, compared to the expected Mendelian inheritance of 50% [1, 7–9]. The principle behind the technology is to re-program a nuclease to cleave a specific site of interest in the genome and to insert the nuclease within this recognition site. The gene drive is designed to be active in the germline, so that in diploid organisms heterozygous for the gene drive the nuclease causes a double stranded break (DSB) at the target site on the homologous chromosome not containing the gene drive. The DSB can be repaired either by simple end-joining (EJ) of the broken strands or via homology-directed repair (HDR) where the DSB is resected and the intact chromosome used as a template to synthesise the intervening sequence. In the case of a gene drive, repair via HDR thus leads to a copying of the drive from one chromosome to another and the conversion of a heterozygote into a homozygote. Hence the force of gene drive is determined by a combination of the rate of cleavage of the nuclease in the germline, and the propensity for the cell machinery to repair the broken chromosome by HDR.

We and others have shown that in germline cells the rates of HDR following a nuclease-induced DSB can be almost two orders of magnitude greater than EJ, a fact which explains the extraordinarily high rates of gene drive inheritance observed [7, 8, 10, 11]. On the other hand EJ repair can lead to the creation of small insertions or deletions at the target site that, although occurring initially at low frequency, might be expected to be selected for in the target organism if they prevent the gene drive nuclease acting and there is a negative fitness cost associated with the gene drive [1, 7, 11–13]. This possibility has been recognised since the first proposal of this type of gene drive [1], with much theory being dedicated to it recently [13, 14] and recent empirical evidence of its occurrence in *Drosophila*[10]. To lower the likelihood of resistance arising there are several potential mitigation strategies including, but not limited to, the targeting of conserved sequences that are less tolerant of mutations and the targeting of multiple sequences, akin to combination therapy [1, 12].

We previously developed a gene drive designed to spread into a mosquito population and at the same time reduce its reproductive potential by disrupting a gene essential for female fertility, thus imposing a strong fitness load on the population [11]. To investigate the long term dynamics of the emergence of resistance to a gene drive imposing such a load we continued to monitor the frequency of this gene drive over generations and analysed the target locus for evidence of mutagenic activity that could lead to the development of resistant alleles that block gene drive activity and restore gene functionality. Our findings show that a range of different resistant alleles can be generated and some of these are subsequently selected for and show dynamics consistent with our modelling predictions. These results provide a quantitative framework for understanding the dynamics of resistance in a multi-generational setting and allow us to make recommendations for the improvement of future gene drive constructs that relate to choice of target site and regulation of nuclease expression in order to retard the emergence of resistance.

## Results

### Long term dynamics of spread of a population suppression gene drive in a caged mosquito population

**Fig 1:**
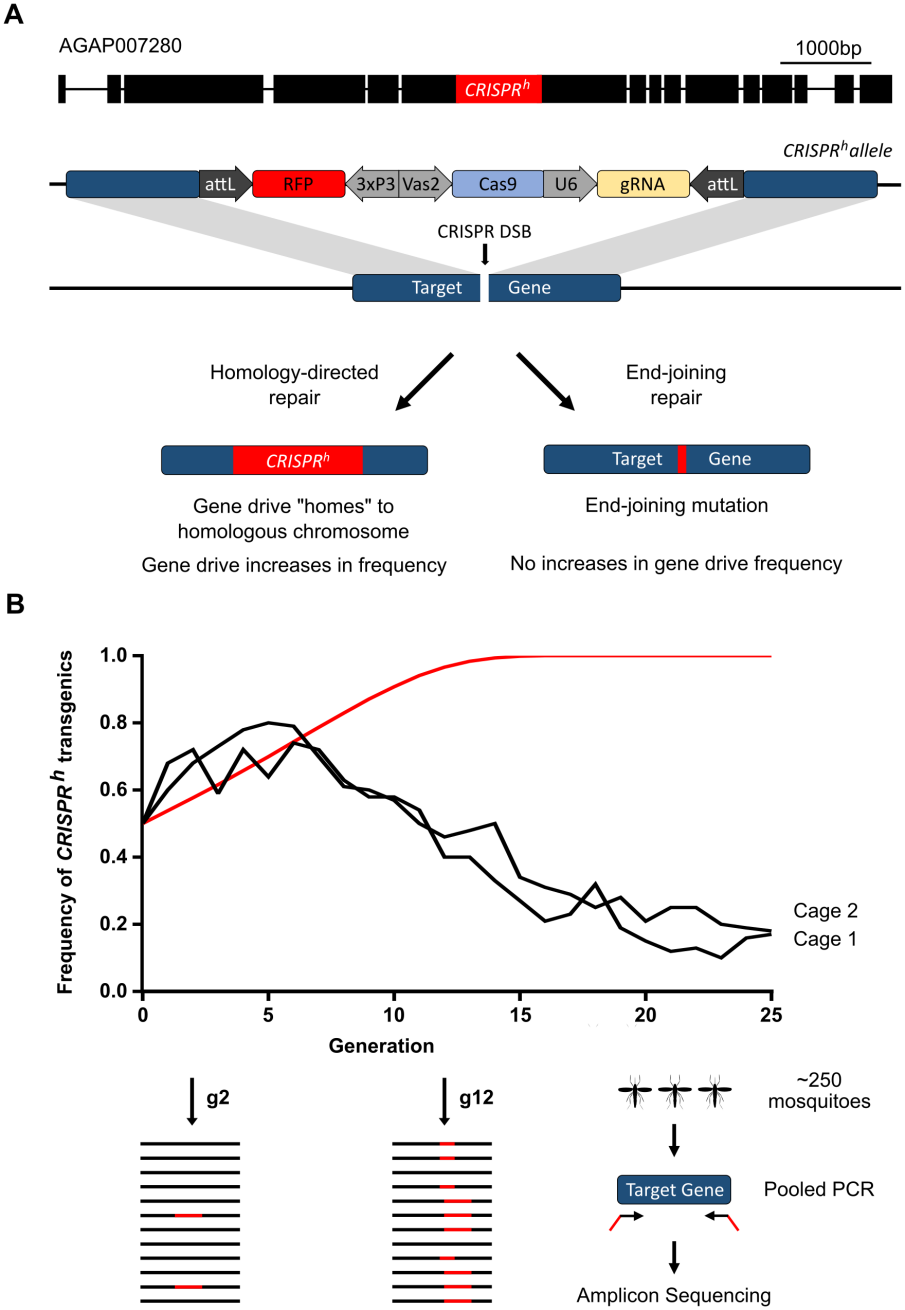
Dynamics of a population suppression gene drive construct over 25 generations. **(A)** Design of the CRISPR-based gene drive construct and the relevant position of its target site within AGAP007280; **(B)** The proportion of individuals containing at least one copy of the gene drive in two replicate cages, monitored each generation for 25 generations. Black lines represent the observed frequencies in each of the two cage trials (CT1 and CT2), red line represents the predicted frequency according to the previous deterministic model that did not take into account target site resistance [11]. Samples were taken for pooled PCR and sequencing analysis of the gene drive target site at G_2_ and at G_12_, whereby frequency of target site indels (shown figuratively as red bars) were revealed by their relative representation among the sequenced reads.

A proof-of-principle CRISPR-based gene drive designed for population suppression was previously developed in our laboratory (Fig 1A). This gene drive disrupted a haplosufficient gene (AGAP007280, the putative mosquito ortholog of *nudel* [15]) required in the soma and essential for female fertility [11]. The gene drive also contained an RFP marker gene for the visual detection of individuals inheriting the drive. In our experiments individuals heterozygous for the gene drive transmitted the drive, regardless of their sex, to more than 99% of their offspring. We observed in these mosquitoes a marked reduction in fertility (~90%) in females heterozygous for the drive, due to ectopic expression of the nuclease under control of the germline *vasa2* promoter that resulted in conversion to the null phenotype in somatic cells. In spite of this fitness disadvantage experimental data showed that the gene drive could increase rapidly in frequency in a caged population due to the exceptionally high rates of inheritance bias. From a starting population (G_0_) in two duplicate cages of 600 individuals with a 1:1 ratio of transgenic heterozygotes and wild type individuals, the gene drive progressively increased in frequency to 72–77% by G_4_. This rate of increase was slightly higher than predicted by a deterministic model but within the limits of stochastic variation expected [11]. Due to a combination of the partial dominance of the sterility phenotype in heterozygous females and the previously documented generation of target site mutations conferring resistance to the gene drive [11], this first gene drive was not expected to maintain high levels of invasion. Nonetheless it represented a useful experimental model to investigate the long term dynamics of the *de novo* generation of target site mutations and their selection at the expense of a gene drive imposing a large reproductive load. We therefore maintained this cage experiment for 25 generations and used the presence of the RFP marker in the gene drive construct as a proxy to estimate the frequency of individuals containing it. The frequency of gene drive progressively increased in both cages, peaking at around generation 6, and thereafter we observed a gradual and continuing decrease such that by G_25_ the frequency of individuals with the gene drive was less than 20%.

### Sequencing the gene drive target site revealed nuclease-induced mutations

**Fig 2:**
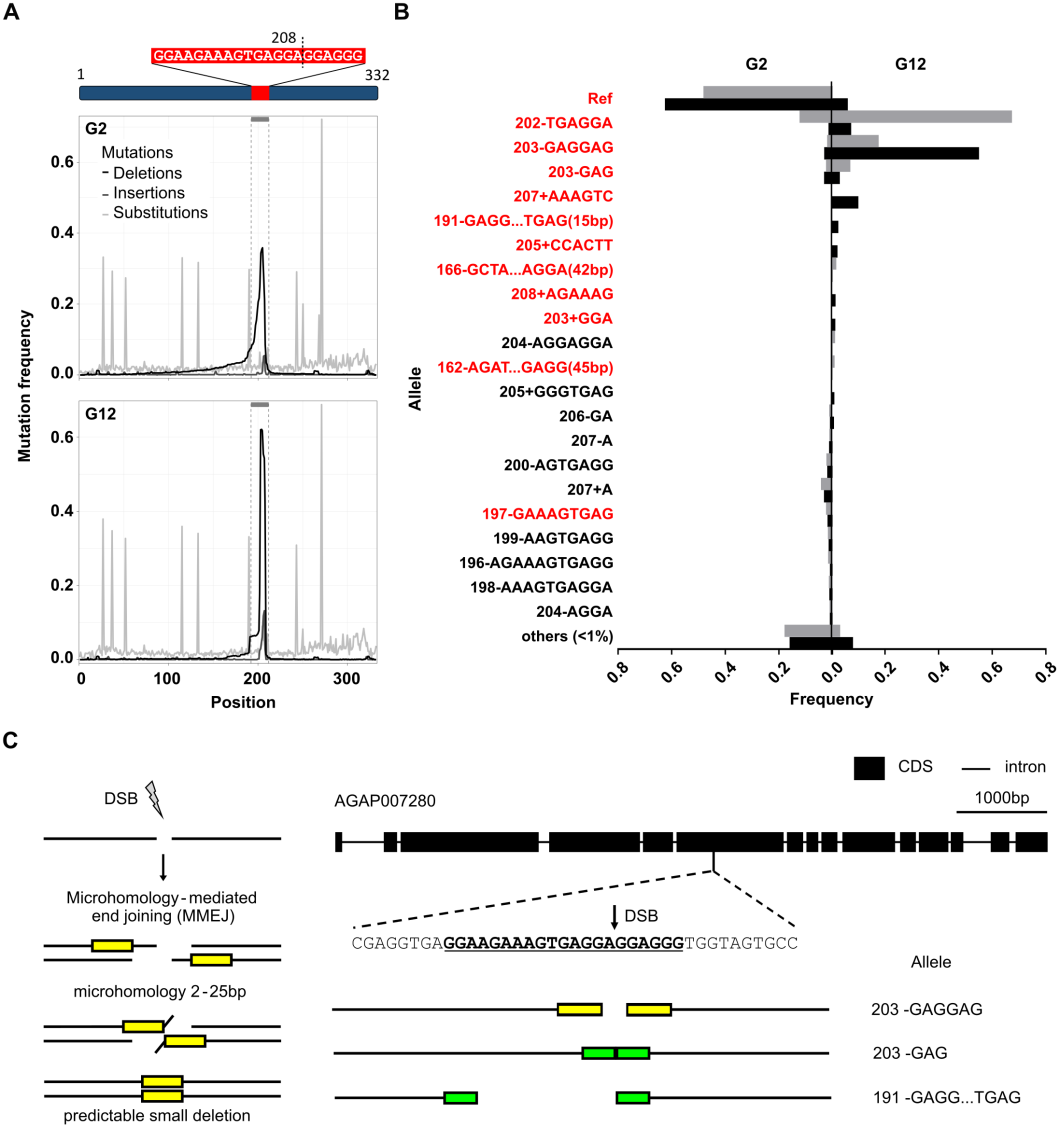
Nuclease-generated target site mutations show selection over time. **(A)** Location of the target site of the CRISPR-based gene drive relative to the region amplified for pooled sequencing (highlighted in red), with the double stranded break occurring after nucleotide 208. Cumulative frequencies of deletions (black), insertions (dark grey) and substitutions (light grey) at each nucleotide position along the amplicon were plotted. Numerous substitutions outside of the target site represent SNPs circulating in the lab strain of mosquito in which the gene drive was introduced and were used for analysis of haplotypes of target site mutations (S1 Fig). One cage trial (CT1) shown as a representative example. **(B)** Frequency of individual target site alleles in both G_2_ and G_12_ for cage 1 (black) and cage 2 (grey) with in-frame indels highlighted in red. All alleles never exceeding 1% frequency in at least one condition were grouped together as one class (<1%). **(C)** Microhomologies flanking the double stranded break at the target could explain some of the most frequent deletions observed. Microhomologies of GAGG (yellow) or GGA (green) and the resultant deletion alleles arising in the event of MMEJ are shown.

To investigate whether the gradual decline in the gene drive frequency observed in the cage experiment was due to the selection of pre-existing variant target sites in the population or the generation and selection of nuclease-resistant indels, we used deep sequencing of a PCR amplicon comprising sequences flanking the target site on pooled samples of mosquitoes from early (G_2_) and late (G_12_) generational time points (Fig 1B). The expected amplified region from the original wild type sequence was 320bp long, with the putative cleavage point within the target site residing after nucleotide 208 (Fig 2A). Ultra-deep sequencing of PCR reactions were performed on pooled DNA under non-saturating conditions so that the number of reads corresponding to a particular allele at the target site is proportional to its representation in the pool. We developed a computational method to analyse the sequences edited by the CRISPR-based gene drive close to the nuclease target site. In the colony of mosquitoes that we used there are a number of pre-existing single nucleotide polymorphisms (SNPs) within the amplicon that do not overlap the nuclease target site and are present at varying frequencies (S1 Fig). Our method identified small insertions and deletions introduced by the repair system and used the presence of surrounding SNPs to characterize the haplotypes on which they arose. Because the PCR only amplifies the non-drive allele, the frequencies reported below refer to their frequency within this class, rather than within the population as a whole.

Mapping amplicon sequences reconstructed from the sequenced against the *Anopheles gambiae* reference genome (PEST strain, AgamP4, Vectorbase) we observed a large repertoire of deletions already in the G_2_ generation, with a wide range of sizes and centred around the predicted nuclease cleavage site after nucleotide 208 (Fig 2A—one cage trial shown as a representative example), and a lower proportion of small insertions, consistent with the known mutational activity of the nuclease. By contrast, ten generations later we observed a much reduced diversity of indels. We then considered all alleles that reached a frequency of at least 1% in any sample, classified these as to whether the indel caused a frameshift in the coding sequence of the target gene or was in-frame, and analysed their frequency over time (Fig 2B). The predominant target allele in the G_2_ was still the reference (non-mutated) allele at 63% and 48% in cages 1 and 2, respectively (Fig 2B and S1 Table), while the second major class (at least 15% in each replicate) was represented by a wide range of non-reference alleles, each present at low frequency (<1%), consistent with the stochastic generation of a broad range of indels. Thus at a time when the gene drive was still increasing in frequency there was a significant accumulation of mutations at the target site that would likely render it refractory to the homing mechanism of copying. Of note, three separate indels causing in-frame deletions of 3- or 6bp (202-TGAGGA, 203-GAGGAG, 203-GAG; where 203 refers to the starting site of the indel in the reference amplicon and “-”means deletion) were present among a large number of indels at low but appreciable frequencies in the G_2_. Such short in-frame deletions may result in only minimal disruption to the final encoded protein while at the same time proving resistant to the gene drive. Indeed these three deletions, plus a 6bp in frame insertion (207+AAAGTC), had increased significantly in frequency to make up the 4 most abundant non-drive alleles in the G_12_, almost to the exclusion of the reference allele (present at 6% and 0.4%; S1 Table). At the same time, a wide range of frameshift indels that were present in G_2_ had fallen in frequency in G_12_ to either below the 1% threshold or were not detected at all (Fig 2B and S1 Table). The most parsimonious explanation for these results is that a wide range of frameshift and in-frame indels was created by the gene drive, yet only short in-frame indels were selected for because they restore functionality to the target gene while protecting the sequence from gene drive activity. These ‘restorative’ mutations are likely to be most strongly selected when the frequency of the gene drive is high in the population—when the majority of individuals are homozygous for the driver, the relative gain in viable offspring from an individual with a gene drive balanced by a resistant restorative mutation is that much higher.

Small in-frame deletions can arise by either classical non-homologous end-joining (NHEJ), or an alternative form of end-joining (microhomology-mediated end-joining, MMEJ) that relies on alignment of small regions of microhomology, as little as 2 base pairs, on either side of the DSB, resulting in loss of the intervening sequence [16]. Consistent with this latter possibility, at least three of the most frequent alleles in the G_12_ generation can be explained by MMEJ via 3bp repeats (Fig 2C).

To investigate whether the most common indels at G_12_ had single or multiple origins, we used the naturally occurring SNPs in the sequences flanking the recognition sequence. In cage 1 the deletion 203-GAGGAG was present on 10 separate haplotypes in G_12_, with each haplotype being present at ratios broadly similar to their ratios in the starting population (S1 Fig), suggesting that the same deletion was generated at least 10 times independently and that there was no detectable selective advantage to any particular haplotype surrounding the deletion. In cage 2 the predominant allele was 202-TGAGGA (68% of all non-reference alleles), an end-joining deletion that shows no apparent features of a MMEJ event, and was found on 5 separate haplotypes.

### Positively selected target site mutations restore function to the target gene and are resistant to gene drive activity

**Fig 3:**
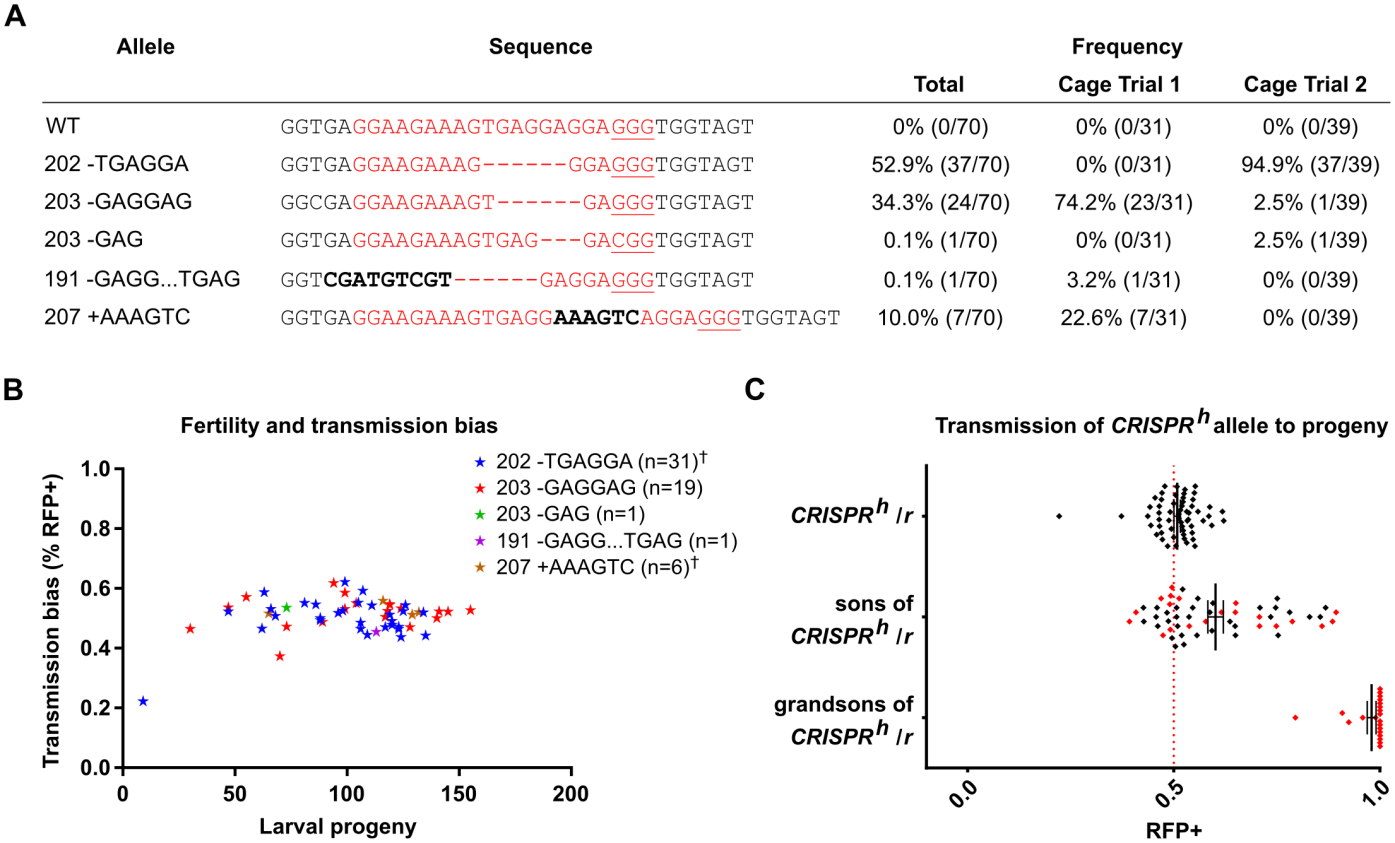
Target site mutations under positive selection are resistant to gene drive activity and restore function to the target female fertility gene. **(A)** Individual females containing at least one copy of the gene drive (RFP+) were selected from the G_20_ generation and the nature of the target allele was determined by PCR and sequencing. Each class of allele is shown with gene drive target sequence highlighted in red and PAM sequence underlined. **(B)** The fecundity of these females and transmission rates of the gene drive were measured and grouped according to allele class at the target site. **(C)** Each *CRISPR*^*h*^*/r* female was used to form a separate lineage and transmission of the gene drive was assessed in sons receiving a maternal copy of the gene drive. A smaller fraction of grandsons receiving a paternal copy of the gene drive were similarly assessed for gene drive transmission. Individual lineages assessed in all three generations are marked in red. † Of 58 mated females one (with deletion 207+AAAGTC) failed to produce eggs while another (202–TGAGGA) produced eggs that failed to hatch.

The progressive increase in frequency of specific mutations at the target site, concomitant with a decrease of gene drive activity, strongly suggested that they conferred resistance to cleavage while still ensuring a normal functional activity of *nudel*. To confirm this hypothesis we crossed individual RFP+ females from G_20_ with wild type males and assessed both their fertility and the transmission rate of the drive. We also sequenced the target site of each parental female to characterize allelic variants at the target locus. This analysis failed to detect wild type sequence at the target site (Fig 3A) among 70 individuals tested; instead every individual showed an indel, indicating that each female tested was heterozygous for the gene drive and balanced by a mutated target site. In cage 1 the 203-GAGGAG 6bp deletion was the predominant allele (23/31 individuals) while in cage 2 another 6bp deletion (202-TGAGGA) was predominant (37/39 individuals). The relative frequency of each allele was consistent with the results obtained using pooled amplicon sequencing performed on the G_12_ individuals.

Of those heterozygous females that could be confirmed as having mated, the vast majority (56/58) generated viable progeny (average clutch size 119 +/- 35.9 eggs, average hatching rate 78.5% +/- 19.9%; Fig 3B and S2 Table) at rates significantly higher than those previously observed in females heterozygous for the gene drive and a wild type allele (90.7% overall reduction in fecundity) [11], suggesting that the mutations detected at the target site substantially restored the functionality of *nudel*. There was no obvious difference in fertility associated with the 5 indels sampled in this assay (Fig 3B). The two major indels across the two cages result in relatively conservative changes to the overall amino acid sequence of the final gene product—two glutamate residues missing (203-GAGGAG) or two glutamates missing and a conversion of a serine to arginine (202-TGAGGA). Finally, the offspring of these individuals showed a non-biased inheritance of the gene drive (50.82% mean +/-5.96% standard error; total RFP+ offspring 50.85%), consistent with normal Mendelian segregation (Fig 3B). Thus, as well as restoring *nudel* function, the mutated sequences were also resistant to the gene drive.

### End-joining repair in the embryo by maternally deposited Cas9 nuclease dramatically increases the generation rate of drive resistant alleles

Conceivably a breakdown in the nuclease component (e.g. mutation in the Cas9 coding sequence, or the gRNA sequence) could be an additional explanation for the Mendelian transmission of the gene drive element and restored fertility in heterozygous females that we observed. To assess this possibility we took the male offspring (‘sons’) of the above crosses that inherited the construct and crossed them in turn to wild type females. We assumed that if the gene drive construct was still functional it should show a biased inheritance when the resistant target site allele had been replaced with a wild type one. Indeed, in these sons we saw a significant increase in the transmission of the gene drive to their progeny, but the observed rate (mean 60.13% +/- 13.9% S.E.; total RFP+ progeny 59.6%) was much lower than that previously observed (~99% inheritance) [11]. A similar phenomenon of reduced homing has been observed in the offspring of another mosquito species [8], and more recently in *Drosophila* [10], when the drive construct was inherited from the mother and when the same *vasa* germline promoter was used to transcribe the Cas9 nuclease. The reduced gene drive activity in the immediate offspring of heterozygous mothers was attributed to the persistence of maternally-deposited Cas9 in fertilized embryos, leading to double stranded DNA breaks being repaired preferentially by end-joining mechanisms in the early zygote possibly before paternal and maternal homologous chromosomes are aligned. Consistent with this explanation, males in the subsequent generation (‘grandsons’) that had received only a paternal copy of the gene drive had exceptionally high homing rates, with 97.5% of progeny inheriting the gene drive (Fig 3C). The drop in homing seen in sons receiving a maternal dose of Cas9 (59.6% transmission vs. 97.5% in grandsons) allows us to estimate an ‘embryonic end-joining’ rate of 79.6% of wild type alleles being converted to cleavage-resistant alleles. This rate of embryonic end-joining is much higher than that observed in the germline at or just prior to meiosis (~1% [11]) and is predicted to reduce the rate of spread of the gene drive, due to a reduced frequency of cleavable alleles [17], and increase the rate at which restorative resistant alleles can arise and be selected.

Cleavage due to maternally deposited Cas9 could potentially be followed by HDR instead of end-joining, effectively leading to ‘embryonic homing’, where the cleaved allele is converted to the non-cleaved allele. In the case of a resistant allele this could lead to an individual heterozygous for the allele being converted into a homozygote in the early zygote, thereby further accelerating the spread of the resistant allele in the population. One signature of embryonic homing of a resistant allele would be novel hybrid haplotypes due to partial conversion of the haplotype surrounding the wild type allele where the DSB was generated to the haplotype surrounding the resistant allele. Looking in detail at the most abundant resistant allele in each cage, we failed to observe such a signature, and all resistant haplotypes were already pre-existing in the population (S1 Fig), suggesting that if this phenomenon is occurring then the resection following cleavage and resultant conversion encompasses a section longer than the ~300bp covered in our sequenced amplicon.

### Incorporating experimental rates of resistance generation into a population model

**Fig 4:**
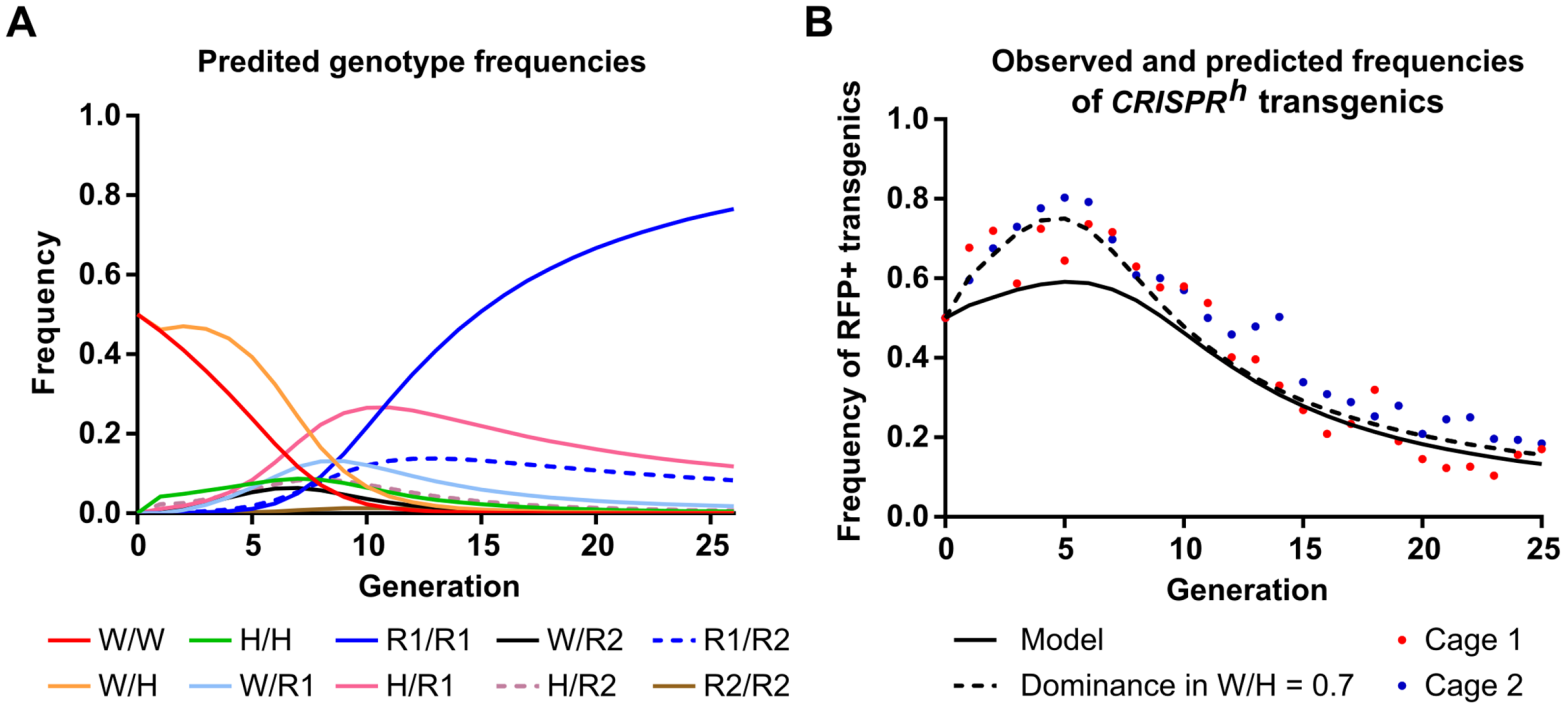
Comparison of observed data with model predicting frequencies of gene drive and resistance alleles. **(A)** Expected genotype frequencies according to the model described in the text and considering the four following target site alleles: wild type (w), *CRISPR^h^* gene drive (h), resistant and in-frame (r1), resistant and out of frame (r2). We used our best experimental estimates of the considered parameters: homing rate (*e*) as 0.984, the dominance of the fertility effect due to leaky somatic expression in females heterozygous (*w/h*) for the gene drive as 0.907, meiotic end-joining rate (γ_m_) as 0.01, embryonic end-joining rate (γ_e_) as 0.796. **(B)** Our observed gene drive frequencies were compared against model predictions using our best experimental estimates (solid black line) and using the best-fit value (0.70 cf 0.907) for dominance of the heterozygous fertility effect in females (dashed black line).

The key qualitative results from the cage experiments—that a gene drive can increase in frequency in a susceptible population even if it reduces individual fitness, and that the spread of a gene drive can in turn lead to the spread of mutants that are resistant to cleavage and restore individual fitness—are fully consistent with expectations from population genetic models [13, 18, 19]. To investigate how well such models can account for the quantitative details of the cage experiments, we extended the model of Deredec *et al*. [18] to incorporate our observations of embryonic cleavage by maternally derived nuclease (which is independent of inheritance of the gene drive), and have two classes of resistant allele (in-frame functional and frame-shift non-functional; see S1 Text and S1 File). Due to the sex-specific fitness effects of our construct, the model also has a separate treatment of females and males. Using this model and the baseline parameter values from the single-generation crosses, we generated the expected allele frequency dynamics over the 25 generations of the experiment (Fig 4). Again, the qualitative fit to the observed dynamics is good, but there are quantitative differences. For example, the model predicts that at G_12_ the original wild type allele will be 9.3% of all non-drive alleles, while our observed rates were 6% and 0.4% in cages 1 and 2, respectively (Fig 2B, S1 Table). The model also recapitulates the observation that while non-functional resistant alleles initially outnumber the functional ones, because they are produced more frequently, by the end of the experiment it is the functional ones that predominate. Importantly, for the gene drive itself, the model captures the essential aspects of the observed dynamics, showing an initial increase in frequency followed by an eventual loss, though in earlier generations our observed frequencies exceeded the predicted frequencies (Fig 4B). To investigate what might explain this discrepancy, we examined the effect of varying each of the different parameter values individually in the model, and found that small variations in the fertility of females heterozygous for the gene drive had the largest effect in increasing the match between observations and expectations. Keeping the experimental estimates of all other parameters unchanged, the least squares best fit occurred at a dominance coefficient of 0.70 (Fig 4B and S1 File), compared to our previous direct estimate of 0.9, with lower confidence limit of 0.86 [11]. We also used our model to investigate the potential impact of HDR after embryonic cleavage caused by maternal deposition of Cas9, and found this parameter has little effect on the expected rate of resistance emergence when the rates of meiotic homing are as high as we observe.

## Discussion

We have analyzed the dynamics of a gene drive deliberately designed to impose a fitness load on a population, and characterized the resistant or compensatory mutations which it generated and selected for. As with any control approach aimed at suppressing an organism, ‘push back’ from the target organism is to be expected. One of the advantages of the modular gene drives investigated here is that contingency in planning for and overcoming resistance can be foreseen and built into the system in a number of ways. First, the use of multiple gene drives targeting separate sequences has long been considered an essential pre-requisite for any gene drive intended as a functional vector control tool [19] and the ease with which the guide RNA expression constructs can be multiplexed lends CRISPR-based gene drives this flexibility [12, 20]. Second, it will be useful to target sites at which sequence changes are likely to destroy function. The nuclease target site in the gene AGAP007280 described in this report was not chosen according to any prioritisation based on high levels of sequence conservation that would imply functional constraint—a feature expected to mean that resistant mutations are less likely to restore function of the gene. Clearly the choice of the target should be guided by the extensive genomic data that is now available on sequence conservation both across different *Anopheles* species [21] and within *An*. *gambiae* [22]. This data revealed *a posteriori* that for the target site in AGAP007280 used here there is pre-existing variation in this species at at least 8 of the 20 nucleotides covered by the gRNA. Going forward low tolerance of sequence variation at the target site should be a key criterion for designing a gene drive. Third, our results show that one of the key drivers in the generation of resistant alleles is the nuclease activity itself, followed by end-joining, and a significant proportion of these alleles are created as a result of maternally deposited nuclease in the early zygote where end-joining repair predominates over homology-directed repair. We suggest this maternal effect may be suppressed either through the use of more tightly regulated promoters to restrict nuclease expression to the early germline or through the addition of destabilising modifications to the nuclease, either of which are expected to reduce perdurance in the embryo. Fourth, an additional consideration in the choice of target site may take into account the propensity for a particular double strand break to be repaired more readily into a resistant, restorative (R2) allele, for example due to microhomology either side of the cleavage site that more readily re-creates an in-frame allele than a frameshift allele. Where MMEJ is the predominant end-joining repair pathway, this feature could be incorporated into target site choice to ensure the most likely end-joining repair event is an out of frame allele and therefore not likely to be selected.

Our approach of pooled sequencing of a targeted region allowed us to reliably detect even low frequency signatures of gene drive activity and reveal the complex dynamics of different genotypes emerging over time. Certainly for the future improvement of gene drives it will be important to have a faster method to triage for the most robust gene drives least prone to resistance without a multi-generational cage experiment, a laborious and time consuming process that should be reserved for more extensive evaluation of the best candidates. A simple way to do this would be to apply the method of amplicon sequencing described here in a screen where all generated mutant alleles are balanced against a known null allele to see if they restore function to the target gene.

The potential for rapid emergence and spread of resistance highlights not only one of the technical challenges associated with developing a gene drive, but also how intentionally releasing a resistant allele in to a population could be a simple and effective means of reversing the effects of a gene drive, if it fully restores function [23].

## Methods

### Gene drive multi-generation cage experiments

These experiments were essentially as described before in Hammond *et al*. [11] Briefly, in the starting generation (G_0_) L1 mosquito larvae heterozygous for the *CRISPR^h^* allele at AGAP007280 were mixed within 12 hours of eclosion with an equal number of age-matched wild-type larvae in rearing trays at a density of 200 per tray (in approx. 1L rearing water). The mixed population was used to seed two starting cages with 600 adult mosquitoes each. For subsequent generations, each cage was fed after 5 days of mating, and an egg bowl placed in the cage 48h post bloodmeal to allow overnight oviposition. After allowing full eclosion a random sample of offspring were scored under fluorescence microscopy for the presence or absence of the RFP-linked *CRISPR*^*h*^ allele, then reared together in the same trays and 600 were used to populate the next generation. After a generation had been allowed the opportunity to oviposit, a minimum of 240 adults were removed and stored frozen for subsequent DNA analysis.

### PCR of target site and deep sequencing library preparation

For the sequence analysis, a minimum of 240 adult mosquitoes taken at generations G_2_ and G_12_ of the cage trial experiments were pooled and extracted en masse using the Wizard Genomic DNA purification kit (Promega). A 332 bp locus containing the target site was amplified from 40 ng of genomic material from each pooled sample using the KAPA HiFi HotStart Ready Mix PCR kit (Kapa Biosystems), in 50 μl reactions. Specially designed primers that carried the Illumina Nextera Transposase Adapters (underlined), 7280-Illumina-F (TCGTCGGCAGCGTCAGATGTGTATAAGAGACAGGGAGAAGGTAAATGCGCCAC) and 7280-Illumina-R (GTCTCGTGGGCTCGGAGATGTGTATAAGAGACAGGCGCTTCTACACTCGCTTCT) were used to tag the amplicon for subsequent library preparation and sequencing. The annealing temperature and time were adjusted to 68°C for 20 seconds to minimize off-target amplification. In order to maintain the proportion of the reads corresponding to particular alleles at the target site, the PCR reactions were performed under non-saturating conditions and thus they were allowed to run for 20 cycles before 25 μl were removed and stored at -20°C. The remnant 25 μl were run for an additional 20 cycles and used to verify the amplification on an agarose gel. The non-saturated samples were used to prepare libraries according to the Illumina 16S Metagenomic Sequencing Library Preparation protocol (Part # 15044223 Rev.A). Amplicons were then purified with AMPure XP beads (Beckman Coulter) followed by a second PCR amplification step with dual indices and Illumina sequencing adapters using the Nextera XT Index Kit.

After PCR clean-up via AMPure XP beads and validation performed with Agilent Bioanalyzer 2100, the normalized libraries were pooled and loaded at a concentration of 10 pM on Illumina Nano flowcell v2 and sequenced using the Illumina MiSeq instrument with a 2x250bp paired-end run.

### Deep sequencing analysis

Sequencing data of the amplified genomic region were analysed using available tools and developed scripts in R v3.3.1. Raw reads were cleaned up for low quality and trimmed for the presence of adapters using Trimmomatic v.0.36 [24].

Paired-end reads were merged together in order to reconstruct the whole amplicon sequence using PEAR v0.9.10 [25]. Resulting assembled identical fragments were then clustered using fastx_collapser module from the FASTX v0.0.13 suite (http://hannonlab.cshl.edu/fastx_toolkit/) and aligned to the reference amplicon with vsearch tool v2.0.3 [26]which implements a global alignment based on the full dynamic programming Needleman-Wunsch algorithm. We considered for downstream analysis only sequences represented by at least 100 reads in each dataset. The blast6 output files from the alignment phase were parsed by ad hoc written R scripts to identify sequence variants containing insertions and/or deletions in the target site. The quantification of each allelic variant was measured as relative alternative allele frequency by summing up the reads representing that particular variant in the dataset. Finally, for each identified variant, we examined the single nucleotide variants (SNVs) along the full amplicon and selected the ones with a minimum alternative allele frequency of 2.5% for the purposes of haplotype calling.

### Haplotype estimation for *A*. *gambiae* G3 laboratory colony

As part of a sequencing effort one year prior to the start of this experiment 12 males and 12 females from our *A*. *gambiae* G3 laboratory colony were subjected to individual genome resequencing. Mosquitoes were chosen randomly as pupae of differing ages from separate trays of a large cohort of the colony population (census population size >2000) in order to minimise biased sampling from a reduced number of founders. After emerging as adults whole mosquitoes were individually homogenised and genomic DNA was extracted with Promega Wizard Genomic DNA extraction kit. Paired-end reads (2 x 100bp) obtained from the Illumina HiSeq 2000 sequencing were aligned to *A*. *gambiae* PEST reference genome assembly (AgamP4, VectorBase) using BWA-MEM (Li and Durbin 2009, v0.7.15). Alignments were sorted using Samtools (v1.5) and raw SNPs and indels were called using HaplotypeCaller tool from Genome Analysis Toolkit (GATK, v3.7) for each of the 24 samples in the same 320bp region around the nuclease target site in AGAP007280 gene that was used for the pooled amplicon sequencing. Raw SNPs were then merged using GATK GenotypeGVCFs tool. No indels were observed in this step for the selected region. We used SHAPEIT2 (Delaneau et al. 2013, v2) for the final haplotype estimation from previously obtained unphased genotypes of 24 individuals. Raw sequencing reads from the 24 individuals have been submited to NCBI Sequence Read Archive (SRA) under project accession number PRJNA397539.

### Modelling

We use a discrete generation deterministic model to explore how the dynamics of gene-frequencies depend on underlying parameters. We suppose there are four possible alleles: Wildtype (W), driver allele (H), and two mutant alleles that are resistant to homing, R1 which is fully functional and R2 which is recessive but non-functional (i.e., H/R2 and R2/R2 type females are sterile). We assume alleles segregate at meiosis according to Mendelian inheritance except in W/H males and females, where segregation may be distorted by cleavage followed by either homing or non-homologous repair. Our model also allows for the possibility that eggs from females with at least one H allele will contain the driver nuclease (regardless of the egg’s own genotype), in which case cleavage and repair may occur in the embryo. The mathematical details of the model are given in the S1 Text, and model outputs from user-defined parameters values can be seen using a computable document format (Wolfram CDF Player) available as a file(S1 File). Baseline parameter values for the model are provided in the legend accompanying Fig 4.

### Individual assays of mosquito fertility and transmission of gene drive

Individual females containing at least one copy of the RFP-linked *CRISPR*^*h*^ gene drive were selected as virgins from the G_20_ generation and allowed to mate with 5 wild type male mosquitoes, essentially as in Hammond *et al*. [11]. The fecundity of females and transmission of the gene drive was measured by counting larval offspring positive for the RFP marker. To check mating status of females, spermathecae were dissected and examined for the presence of sperm. Unmated females were censored from the fertility assay.

Sons of each gene drive mother from the G_20_ generation were kept together and allowed to mate in groups of approximately 5 males with an equal number of wild type females and assessed for rates of transmission of the gene drive. The male offspring of these sons (grandsons) inheriting the drive from their fathers were in turn assessed in the same way, keeping lineages separate.

## Supporting information

S1 Fig Resistant target site alleles showing selection are formed independently on a wide range of haplotype backgrounds. The presence of polymorphic SNPs surrounding the target site and circulating at various frequencies in the laboratory wild type colony allowed us the resolution to identify a variety of haplotypes on which target site indels may have been formed. The most prominent target site indel in each cage replicate in the G_12_ generation was analysed and the number of haplotypes containing the respective indel and the frequency of each haplotype was calculated. A measure of the diversity of pre-existing reference haplotypes present in the colony was obtained by examining the nature and frequency of known haplotypes in the original wild type colony based on resequencing of 24 individuals (48 haplotypes) and the haplotypes surrounding the wild type target site allele in the early G_2_ generation of the cage experiment. In both replicates there were no unique haplotypes containing the indel that were not already pre-existing in the starting population. The relative frequency of haplotypes surrounding a given target site allele are also displayed.
(TIF)

S1 Table Target site allele frequencies at G_2_ and G_12_ generations. (XLSX)

S2 Table Gene drive transmission in offspring of *CRISPR*^*h*^*/R* receiving a maternal copy of gene drive, and subsequent paternal copies. Individual females containing at least one copy of the RFP-linked *CRISPR*^*h*^ gene drive were selected from the G_20_ generation and allowed to mate with 5 wild type male mosquitoes. Offspring were kept as separate lineages and sons and grandsons were usually mated as groups of at least 5 with wild type females. Transmission of the gene drive was monitored by screening for the presence of the linked RFP gene. The target site allele present in the initial G20 female founder of each lineage is also displayed. Summary data is shown on the left, data for each cage shown on the right.
(XLSX)

S1 Text Mathematical details of the model used to predict gene drive dynamics. (PDF)

S1 File Computable document format (Wolfram) allowing user-entered values of parameters that may affect gene drive performance. (CDF)
