# Supplementary figures and images for: The creation and selection of mutations resistant to a gene drive over multiple generations in the malaria mosquito
Source: PLoS Genet. 2017 Oct 4;13(10):e1007039. doi: 10.1371/journal.pgen.1007039 (PMC5648257; doi:10.1371/journal.pgen.1007039)

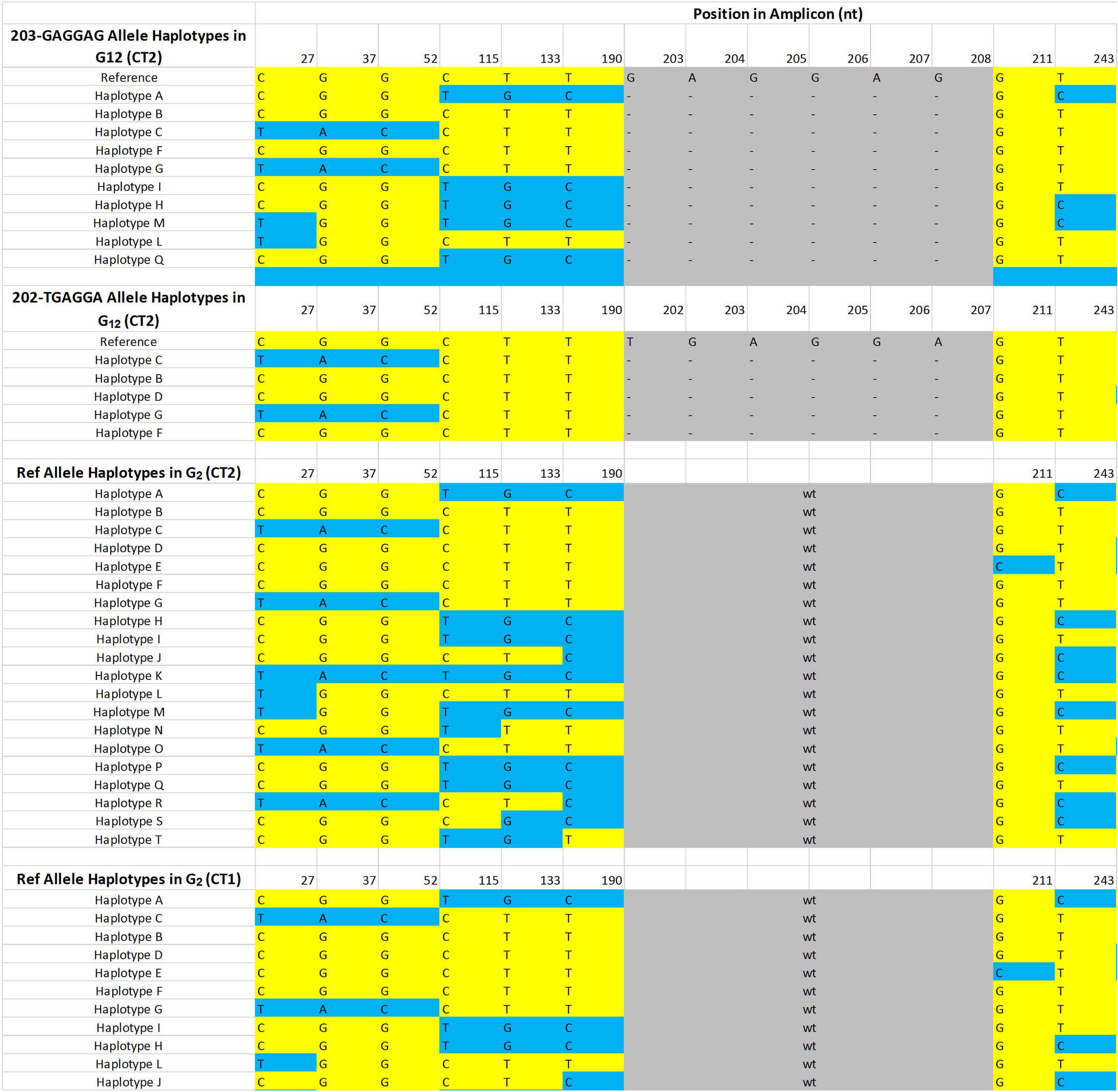

Supplement: S1 Fig — The presence of polymorphic SNPs surrounding the target site and circulating at various frequencies in the laboratory wild type colony allowed us the resolution to identify a variety of haplotypes on which target site indels may have been formed. The most prominent target site indel in each cage replicate in the G12 generation was analysed and the number of haplotypes containing the respective indel and the frequency of each haplotype was calculated. A measure of the diversity of pre-existing reference haplotypes present in the colony was obtained by examining the nature and frequency of known haplotypes in the original wild type colony based on resequencing of 24 individuals (48 haplotypes) and the haplotypes surrounding the wild type target site allele in the early G2 generation of the cage experiment. In both replicates there were no unique haplotypes containing the indel that were not already pre-existing in the starting population. The relative frequency of haplotypes surrounding a given target site allele are also displayed. (TIF) [file pgen.1007039.s001.tif]
