# Supplementary material for: The creation and selection of mutations resistant to a gene drive over multiple generations in the malaria mosquito
Source: PLoS Genet. 2017 Oct 4;13(10):e1007039. doi: 10.1371/journal.pgen.1007039 (PMC5648257; doi:10.1371/journal.pgen.1007039)
Supplement: S1 Text — (PDF) [file pgen.1007039.s004.pdf]

Population genetics of a homing endonuclease and resistant alleles

Genetic model

We consider a homing allele 'H' inserted at a locus that is normally occupied by a wildtype allele 'W'. Further, mutant alleles R_1 and R_2 (that are resistant to homing) may establish at this locus either from an NHEJ (or MMEJ) event or from a low frequency in the standing genetic variation. The model is based on models developed by Deredec et al. (2008) and Unckless et al. (2017), which we tailored to correspond to our specific construct. We note that in the absence of resistance alleles, our model distils to exactly that of Deredec et al. (2008) (their equation 15) if, in their model, it is assumed that females are fully sterile and males fully fit ($s_f = 1$ and $s_m = 0$ in their notation), homing occurs equally in each sex (e_m=e_f in their notation), and allele frequencies are in Hardy-Weinberg equilibrium. In this case Deredec et al. show that the driver gene will spread from rare to an equilibrium frequency in coexistence with the wildtype allele (eqn. 16 of Deredec et al., 2008).

Genotype fitness assumptions

The wildtype gene is involved in female fertility so that H/H females are fully sterile and W/H females have fecundity $(1 - h)$ relative to wildtype females (the parameter h thus defines the dominance of the homing allele in W/H heterozygotes). We suppose R_1 is a functional resistant allele while R_2 is non-functional. Specifically, we assume females with the R_1 allele have fertility $1 - s$ when paired with a non-functional (H or R_2) homologue or itself (the parameter s thus defines the R_1 allele fitness cost), while the R_2 allele is non-functional but recessive (i.e. R_1/R_2 and W/R_2 are functional, but females that are H/R_2 and R_2/R_2 are sterile). Although it is possible that the genotype of an individual will also impinge on viability and other fitness traits, we have no experimental evidence for this and thus assume that only female fertility is affected. We thus do not need to consider different mosquito life-stages, as adult genotypic frequencies are equivalent to zygote frequencies.

Nuclease expression

Meiotic expression

We suppose that adults (both male and female) with the genotype W/H create gametes at meiosis in the ratio $\theta : e'_m : \gamma'_{m_1} : \gamma'_{m_2}$ of $W : H : R_1 : R_2$, where

$$
\begin{aligned}
\theta &= \frac{1 - e_m - \gamma_m}{2} \\
e'_m &= \frac{1 + e_m}{2} \\
\gamma'_{m_1} &= \frac{\gamma_m}{6} \\
\gamma'_{m_2} &= \frac{\gamma_m}{3}.
\end{aligned}
$$

Here e_m is the 'meiotic homing rate' and γ_m is the 'meiotic NHEJ rate' that corresponds to the creation of R_1 and R_2 alleles by an NHEJ (or MMEJ) event during meiosis. We suppose these alleles are created in the ratio of 1 : 2 (of $R_1 : R_2$) because we assume all 'multiples of three' base-pair deletions and additions result in R_1 type functional alleles, and all other deletions and additions result in R_2 type non-functional alleles.

Embryonic expression

To allow for the possibility of cleavage and repair occurring in the embryo, we suppose gametes (of all types) produced by X/H females ($X \in \{W, R_1\}$) may contain maternally deposited nuclease. We assume this cleaves W alleles to be repaired by NHEJ with probability γ_e (the 'embryonic NHEJ rate') and by homing with probability e_e (the 'embryonic homing rate'). (The wildtype allele is preserved otherwise, with probability $1 - \gamma_e - e_e$). As per the meiotic case, embryonic NHEJ creates resistance alleles in the ratio 1 : 2 of $R_1 : R_2$. We note that homing may convert W/R_X heterozygotes into R_X/R_X homozygotes as well as driver heterozygotes into homozygotes.

Derivation of model equations

Our model aims to mimic the cage experimental set-up so that we can compare model output to the experimental results. Since the genotypic composition of the first generation in the cage is known, we chose to construct the model in the genotype frequency domain, rather than the allele frequency domain. This formulation also enables incorporation of the maternal effect (embryonic expression of maternally deposited nuclease) in a straightforward manner.

We write G_{XY} as the frequency of genotype X/Y in a given generation and G'_{XY} as the corresponding frequency in the subsequent generation. We seek to derive the mapping $\{G_{XY} \to G'_{XY}\}_{X,Y \in \{W,H,R_1,R_2\}}$. First, we separately derive the frequencies of all sperm and ovum gametes that form the next generation. We denote sperm gametic frequencies as M_W, M_H, M_{R_1}, and M_{R_2}, and the relative ovum gametic frequencies as $F_W, F_H, F_{R_1}, F_{R_2}, F^\dagger_W, F^\dagger_H, F^\dagger_{R_1}$, and $F^\dagger_{R_2}$, where the $\cdot^\dagger$ indicates that the gamete derives from a X/H female, and so contains the nuclease. We have

$$\text{Sperm gametic frequencies:} \quad \begin{cases} M_W : G_{WW} + \theta G_{WH} + \frac{1}{2}G_{WR_1} + \frac{1}{2}G_{WR_2} \\ M_H : G_{HH} + e'_m G_{WH} + \frac{1}{2}G_{HR_1} + \frac{1}{2}G_{HR_2} \\ M_{R_1} : G_{R_1R_1} + \gamma'_{m_1} G_{WH} + \frac{1}{2}G_{WR_1} + \frac{1}{2}G_{HR_1} + \frac{1}{2}G_{R_1R_2} \\ M_{R_2} : G_{R_2R_2} + \gamma'_{m_2} G_{WH} + \frac{1}{2}G_{WR_2} + \frac{1}{2}G_{HR_2} + \frac{1}{2}G_{R_1R_2} \end{cases}$$

$$\text{Ovum relative gametic frequencies:} \quad \begin{cases} F_W : G_{WW} + \frac{1}{2}G_{WR_1} + \frac{1}{2}G_{WR_2} \\ F_H : 0 \\ F_{R_1} : (1-s)G_{R_1R_1} + \frac{1}{2}G_{WR_1} + \frac{1-s}{2}G_{R_1R_2} \\ F_{R_2} : \frac{1}{2}G_{WR_2} + \frac{1-s}{2}G_{R_1R_2} \\ F^\dagger_W : (1-h)\theta G_{WH} \\ F^\dagger_H : (1-h)e'_m G_{WH} + \frac{1-s}{2}G_{HR_1} \\ F^\dagger_{R_1} : (1-h)\gamma'_{m_1} G_{WH} + \frac{1-s}{2}G_{HR_1} \\ F^\dagger_{R_2} : (1-h)\gamma'_{m_2} G_{WH}. \end{cases}$$

We note that the sum of the sperm gametic terms equates to the sum of all genotypic frequencies and so equals one, while the sum of the ovum gametic terms equates to

$$1 - G_{HH} - G_{HR_2} - G_{R_2R_2} - hG_{WH} - s\left(G_{HR_1} + G_{R_1R_1} + G_{R_1R_2}\right)$$

which may be less than one if alleles that reduce fertility are present (hence we refer to these terms as relative frequencies).

To compute the frequencies of each genotype in the next generation we consider the sperm and ovum gametic combinations that give rise to the given genotype, after taking account of the fact that all $F^{\dagger}_X$ type ovum gametes may convert a wildtype allele via NHEJ or homing. We denote the relative frequency of genotype XY in the next generation as G^*_{XY}. The full set of equations for all G^*_{XY} are

$$\begin{aligned}
G^*_{WW} &= M_W(F_W + F^{\dagger}_W(1-\gamma_e)^2) \\
G^*_{WH} &= M_W(F_H + F^{\dagger}_H(1-\gamma_e - e_e)) + M_H(F_W + F^{\dagger}_W(1-\gamma_e - e_e)) \\
G^*_{HH} &= M_H(F_H + F^{\dagger}_H) + e_e M_W F^{\dagger}_H + e_e M_H F^{\dagger}_W \\
G^*_{WR_1} &= M_W(F_{R_1} + F^{\dagger}_{R_1}(1-\gamma_e - e_e) + F^{\dagger}_W 2\gamma_e(1-\gamma_e)/3) + M_{R_1}(F_W + F^{\dagger}_W(1-\gamma_e - e_e)) \\
G^*_{R_1R_1} &= M_{R_1}(F_{R_1} + F^{\dagger}_{R_1} + F^{\dagger}_W\gamma_e/3) + M_W(F^{\dagger}_W(\gamma_e/3)^2 + F^{\dagger}_{R_1}\gamma_e/3) \\
&\quad + e_e M_W F^{\dagger}_{R_1} + e_e F^{\dagger}_W M_{R_1} \\
G^*_{HR_1} &= M_H(F_{R_1} + F^{\dagger}_{R_1} + F^{\dagger}_W\gamma_e/3) + M_{R_1}(F_H + F^{\dagger}_H) + M_W F^{\dagger}_H\gamma_e/3 \\
G^*_{WR_2} &= M_W(F_{R_2} + F^{\dagger}_{R_2}(1-\gamma_e - e_e) + F^{\dagger}_W 2\gamma_e(1-\gamma_e)(2/3)) + M_{R_2}(F_W + F^{\dagger}_W(1-\gamma_e - e_e)) \\
G^*_{HR_2} &= M_H(F_{R_2} + F^{\dagger}_{R_2} + F^{\dagger}_W\gamma_e(2/3)) + M_{R_2}(F_H + F^{\dagger}_H) + M_W F^{\dagger}_H\gamma_e(2/3) \\
G^*_{R_1R_2} &= M_{R_1}(F_{R_2} + F^{\dagger}_{R_2} + F^{\dagger}_W(2/3)\gamma_e) + M_{R_2}(F_{R_1} + F^{\dagger}_{R_1} + F^{\dagger}_W\gamma_e/3) \\
&\quad + M_W F^{\dagger}_{R_1}(2/3)\gamma_e + M_W F^{\dagger}_{R_2}\gamma_e/3 + 2M_W F^{\dagger}_W(\gamma_e/3)(\gamma_e(2/3)) \\
G^*_{R_2R_2} &= M_{R_2}(F_{R_2} + F^{\dagger}_{R_2} + F^{\dagger}_W\gamma_e(2/3)) + M_W(F^{\dagger}_W(\gamma_e(2/3))^2 + F^{\dagger}_{R_2}\gamma_e(2/3)) \\
&\quad + e_e M_W F^{\dagger}_{R_2} + e_e F^{\dagger}_W M_{R_2}.
\end{aligned}$$

Finally, these are converted to the next generation frequencies $\{G'_{XY}\}$ by dividing by the total,

$$\begin{aligned}
G_{Tot} &= G^*_{WW} + G^*_{WH} + G^*_{HH} + G^*_{WR_1} + G^*_{R_1R_1} + G^*_{HR_1} + G^*_{WR_2} + G^*_{HR_2} + G^*_{R_1R_2} + G^*_{R_2R_2} \\
&= \left(F_W + F_H + F_{R_1} + F_{R_2} + F^{\dagger}_W + F^{\dagger}_H + F^{\dagger}_{R_1} + F^{\dagger}_{R_2}\right)(M_W + M_H + M_{R_1} + M_{R_2}) \\
&= 1 - G_{HH} - G_{HR_2} - G_{R_2R_2} - hG_{WH} - s\left(G_{HR_1} + G_{R_1R_1} + G_{R_1R_2}\right),
\end{aligned}$$

giving $G'_{XY} = G^*_{XY}/G_{Tot}$ for all the genotypes X/Y.

References

Deredec, A., A. Burt, and H. C. J. Godfray, 2008. The population genetics of using homing endonuclease genes in vector and pest management. Genetics 179:2013–2026.

Unckless, R. L., A. G. Clark, and P. W. Messer, 2017. Evolution of resistance against crispr/cas9 gene drive. Genetics 205:827–841.
